# A cross-sectional survey on attitudes of men who have sex with men towards anal self-examination for detection of anal syphilis

**DOI:** 10.1038/s41598-022-12881-3

**Published:** 2022-05-27

**Authors:** Ei T. Aung, Christopher K. Fairley, Jason J. Ong, Tiffany R. Phillips, Marcus Y. Chen, Julien Tran, Kate Maddaford, Elena R. Rodriguez, Eric P. F. Chow

**Affiliations:** 1 Melbourne Sexual Health Centre, Alfred Health, 580 Swanston Street, Carlton, VIC 3053 Australia; 2 Central Clinical School, Faculty of Medicine, Nursing and Health Sciences, Monash University, Melbourne, VIC Australia; 3 Centre for Epidemiology and Biostatistics, Melbourne School of Population and Global Health, The University of Melbourne, Melbourne, VIC Australia

**Keywords:** Disease prevention, Preventive medicine, Population screening, Infectious diseases

## Abstract

Studies suggest men who have sex with men (MSM) practising receptive anal sex are more likely to present with secondary syphilis, implying primary anorectal lesions are likely to be missed. If men could detect anorectal lesions in the primary stage by regular anal self-examination (ASE), transmission could be reduced by early diagnosis and treatment. We aimed to explore the attitudes of MSM on performing ASE to detect primary anorectal syphilis. An online anonymous cross-sectional survey among MSM over 18 years of age living in Australia, was conducted between July and November 2020 and recruitment was from a sexual health clinic and social media. A total of 568 MSM completed the survey (median age: 34 [IQR 27–45]): 32% (183) had previously performed ASE. Among 66% (374) who had never performed ASE, 68% (250) would consider performing ASE in the future with a preferred median frequency of 2 times per 4 weeks (IQR 1–4), whilst men who were already performing ASE were performing it at median 1 per 4 weeks (IQR 0.2–3). Almost two-thirds of MSM who had never performed ASE were willing to adopt ASE practice in the future. Studies are required to determine the effectiveness of ASE for syphilis detection.

## Introduction

Syphilis continues to rise among gay, bisexual and other men who have sex with men (MSM) in high-income countries despite numerous public health interventions to improve syphilis prevention and control^1–5^. Previous public health strategies included regular serological screenings of MSM who are considered at a higher risk of acquiring syphilis, improved contact tracing, and behavioural interventions to increase condom use^6–8^. Despite these measures, syphilis cases continue to rise prompting the need for additional strategies for syphilis control^9^.

Reducing the duration of the infectious period is one of the main strategies underpinning interventions for syphilis control. Primary syphilis classically presents as a painless chancre at the point of inoculation^10^. These primary lesions may go unnoticed if they occur at hidden sites such as the vaginal or inside the anal canal. A study examining the shedding of *Treponema pallidum* showed three out of 54 (6%) MSM with primary syphilis had syphilis lesions in the anorectum confirming the presence of primary syphilis lesions at the sites that are often overlooked^11^. A retrospective study in Australia reported that MSM who practised receptive penile–anal sex were four times more likely to present with secondary syphilis than those who practised insertive penile–anal sex, suggesting primary anorectal lesions are often missed^12^. Similar findings were reported in another study where MSM who practised receptive penile–anal intercourse were 48% less likely to be diagnosed in the primary stage than other MSM, implying occult primary lesions are probably undiagnosed^13^. Furthermore, another Australian study on MSM taking PrEP has found that routine scheduled 3-monthly syphilis screening during PrEP visits failed to detect a substantial proportion of infectious syphilis (58% of primary syphilis and 44% of secondary syphilis), indicating that a 3-monthly serological screening alone may be insufficient for syphilis control among this population^14^. Recent studies have shown MSM with secondary syphilis often have *T. pallidum* detection at multiple sites, including the mouth and anus, suggesting that preventing the secondary stage of syphilis may also reduce the infectiousness^11^. Furthermore, early detection of anal syphilis might reduce the duration of infectious primary syphilis lesions, thereby improving the syphilis control. It is hypothesised that if men examine their anus weekly in addition to 3-monthly routine screening, they might be able to detect primary anorectal lesions themselves and present for treatment before progressing to secondary syphilis, thereby reducing infectiousness.

Anal self-examination (ASE) is a new concept for syphilis detection; however this practice has been introduced among MSM aged over 50 who are living with HIV to detect early anal cancer with good acceptability^15–20^. A qualitative study among 20 MSM recruited from a sexual health centre exploring their attitudes towards ASE for anal syphilis, and found that performing ASE was highly acceptable^21^. Past case reports have confirmed the presence of palpable primary syphilis lesion in the anus which was noticed by the patient during self-examination suggesting the potential use of anal self-examination to detect anal syphilis lesions^22^. We designed this online anonymous survey study to further explore the perspectives of MSM recruited both at a sexual health centre and from the general community on ASE practice. This study aimed to investigate the attitudes and perceptions on ASE among MSM and their willingness to adopt the practice routinely for syphilis detection.

## Method

### Study population and distribution

We conducted a cross-sectional anonymous online survey named “Self-Examination of Anal Syphilis—a Survey study” (SEAS-S) between July and November 2020. Men who self-reported having sex with other men, aged 18 and above, and living in Australia were eligible to participate in the survey. Men were recruited from the Melbourne Sexual Health Centre (MHSC), Victoria, Australia during the study period. All men attending MSHC were asked whether they would like to receive additional information (e.g., research studies) via SMS with their registered mobile number. An SMS explaining the SEAS-S study and a link to the online survey was sent to all men who provided consent to receive additional information each day during the study period. Furthermore, men were also recruited from the general population across Australia. A link to the survey was posted and distributed through social media (e.g., Facebook, Twitter) as well as internal social networks with community-based organisations supporting lesbian, gay, bisexual, transgender, intersex, queer people or questioning (LGBTIQ+) communities and people living with HIV in Australia.

Ethical approval was obtained from the Alfred Hospital Ethics Committee, Melbourne, Australia (366/20) and endorsement obtained from Thorne Harbour Health, a community organisation in Victoria, Australia (THH/CREP 20-013). All research was performed in accordance with the relevant guidelines/regulations of the Alfred Hospital Ethic Committee.

Informed consent was obtained from all the participants (see under survey information outlining the process of obtaining informed consent).

### Survey information

The first page of the online survey described the detailed nature of the study, including a participant information sheet that the participants could download. Participants were required to provide consent to participate in the survey by selecting the ‘Agree’ button on the first page before commencing the survey. Participants who decided not to participate could select the ‘Disagree’ button to terminate the survey.

The survey collected demographic characteristics such as age, country of residence, gender, sexual orientation, HIV status and currently taking pre-exposure prophylaxis (PrEP) for HIV, and whether the participants had ever performed ASE. Anal self-examination was defined as inserting a finger into one’s anus, feeling around the anal canal (360°), and using a mirror to check the anus and surrounding area for any abnormalities. This was an anonymous survey, and no other personally identifiable information was collected in the survey.

In our qualitative study which is part of the series of anal examination study for detection of anorectal syphilis^21^, men who reported inserting their fingers for pleasuring themselves noticed abnormal findings during the examination. Therefore, in this study, we only asked about ASE performed for the purpose of detecting abnormalities. Those who had ever performed ASE were asked about the positions, accessories, and locations when they performed ASE, and resources and information likely to be helpful for men who were new to ASE. Participants who had never performed ASE were asked about the possible positions and locations that they would likely use to perform ASE and resources and information that they might find useful to learn ASE.

For analysis purpose, we included surveys with minimum completion of some demographics such as gender, sexual orientation, sex position, education, and HIV and PrEP status, whilst the surveys that did not meet the minimum completion were excluded.

The survey was generated and conducted using the Qualtrics platform (Qualtrics, Provo, UT), which collected the location latitude and longitude automatically where the participant completed the survey. The location latitude and longitude were converted to the location of the city. Participants with a location outside Australia were excluded from this analysis.

### Statistical analysis

Descriptive statistics were computed to summarise the demographic characteristics and study variables. Men were categorised into two groups based on whether they had ever performed ASE: ‘ever performed’ and ‘never performed’. Demographic characteristics between men who had ever performed and those who had never performed ASE were compared using Fisher’s exact test for categorical variables and Mann–Whitney *U* test for continuous variables. Individuals were able to decline to answer any questions in the survey and they were considered as missing data. The proportion of the study variables was calculated based on the number of participants who answered that question excluding those who declined to answer. Some survey questions had more than one options to choose from, and therefore, the answers were not mutually exclusive, and the total might add up to more than 100%. This will be flagged in the tables and figures. All analyses were conducted with STATA (StataCorp. 2019. Stata Statistical Software: Release 16. College Station, TX: StataCorp LLC.).

## Results

Between July and November 2020, a total of 3154 SMS was sent to men attending MSHC. Of those, 620 (20%) clicked the survey link and started the survey. Additionally, 89 surveys were received through social media from the community. Hence, a total of 709 responses were received. We excluded 141 participants, including 77 who did not complete surveys; 33 did not consent to participate the survey; 19 living outside Australia or declined to disclose where they lived; eight reported no sexual contact with another man in the last 12 months; and four self-identified as cis-female or female gender.

The remaining 568 men were included in the final analysis and the median age was 34 (IQR 27–45) (Table 1). The majority were cis male (98%, n = 556). Most men reported sex with men only (91%, n = 514), and a small proportion reported having sex with both men and women (9%, n = 50). There were a few men who preferred not to report their sexual orientation (1%, n = 7). About one-third of men reported they had ever performed ASE (32%, n = 183) among 557 men who answered the question on previous experience of ASE. Table 1 shows the demographic characteristics of men who had performed ASE previously and who had never performed ASE with no statistically significant differences between the two groups.

**Table 1:** Demographics of survey participants.

	Ever performed ASE (N = 183)		Never performed ASE (N = 374)		p-value
Age, median (interquartile range)	35 (IQR 29–47)		33 (IQR 27–43)		0.041
	**Gender**^**1**^				0.630
Male	178	97.0%	365	98.0%	
Non-binary/gender fluid	4	2.0%	5	1.0%	
Other gender	1	0.6%	4	1.0%	
	**Education**				0.126
No post-school qualification^#^	24	13.6%	51	13.3%	
Certificate and diploma* level	43	24.0%	76	20.0%	
University degree^^^	116	64.0%	247	66.0%	
	**Sexual orientation**				0.458
Gay	169	92.0%	334	89.0%	
Bisexual	13	7.0%	35	9.0%	
Prefer not to report	1	0.6%	5	1.0%	
	**Anal sex position**				0.188
Receptive anal sex	63	34%	105	28.0%	
Versatile anal sex	95	52%	193	52.0%	
Insertive anal sex	22	12%	69	18.0%	
No anal or penetrative sex	3	2%	7	2.0%	
	**HIV status and PrEP use**				0.773
Living with HIV	46	25.0%	86	23.0%	
Taking PrEP	71	39.0%	133	36.0%	
Not living with HIV and not taking PrEP	61	33.0%	140	37.0%	
Unknown or prefer not to say	5	3.0%	15	4.0%	
**Previous syphilis infection**	72	39.0%	126	34.0%	0.346

^1^Female was not reported in the table as those identified as female at birth were excluded.

*Certificate* I, II, III, IV level, advanced diploma and diploma level.

^^^Bachelor’s degree, master’s degree/graduate diploma/graduate certificate, postgraduate degree.

^#^No schooling completed, some secondary education—years 9 and below, secondary education completed.

Kruskal–Walis *H* test was used to compare the statistical significance of age between men who had previously performed ASE and men who had never performed ASE. Pearson’s chi test was used to compare the other demographic characteristic between the two groups.

### Preferences for performing self-examination among men who had ever performed ASE

**Figure 1:**
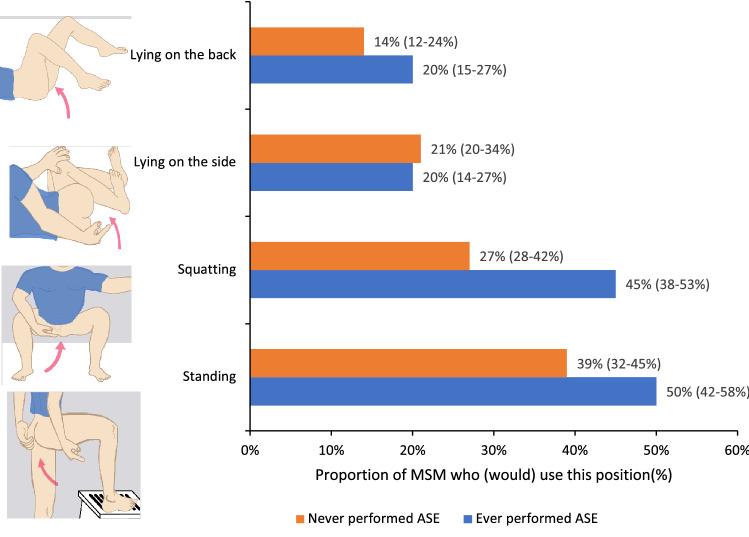
Positions used by MSM who had ever performed ASE and positions likely to be used by MSM who had never performed ASEs. Multiple positions were selected, and the total will exceed 100%. The choices were not mutually exclusive. 95% confidence interval expressed in brackets following the proportion. Note: 176 men who had ever performed ASE and 227 who had never performed ASE answered questions on sexual positions. Six men in the ever-performed ASE chose other positions. Other positions were not asked in the never performed ASE group. This category was not included in the graph.

The median frequency of performing ASE was one (IQR 0.2–3) per four weeks. Of 183 men who had ever performed ASE, 176 provided answers for ASE positions, and the most commonly reported position was standing (50%, n = 88), followed by squatting (45%, n = 80) (Fig. 1). There were 28% (n = 50) men who reported using more than one position for ASE, and the most common combination was standing and lying on their back (5%, n = 8).

Most men performed ASE in the shower (61%, n = 106), followed by in the bathroom or toilet (49%, n = 85). Most men (94%, n = 163) reported using at least one accessory when performing ASE. The most commonly used accessory was lubricant including sorbolene and saliva(59%, n = 103), followed by water (39%,n = 68), soap (26%, n = 45), mirror (24%, n = 42), and gloves (11%, n = 19) during ASE (Table 2).

**Table 2:** Type of abnormalities felt during ASE, ease of performing the examination and likelihood of recommending it to other MSM.

Experience related to ASE	Number of choices	Percentage (%)
**Felt something during ASE**	**N = 173**	
Yes	81	47.0
No	92	53.0
**Description of abnormalities**^^^	**N = 79**	
Lumps	50	63.0
Ulcers or sores	22	28.0
Bleeding	30	38.0
Others *	12	15.0
(Haemorrhoids × 7, warts × 2, tearx1, lump, varicose vein, tenderness)		
**Ease of performing ASE**	**N = 172**	
Very easy	43	25.0
Easy	72	42.0
Neutral	44	26.0
Difficult	11	6.0
Very difficult	2	1.0
**Likelihood of recommending ASE to other men by men who had ever performed ASE**	**N = 167**	
Very likely	74	44.0
Likely	60	36.0
Maybe	21	13.0
Unlikely	9	5.0
Very unlikely	3	2.0

^^^Allowed multiple options and the answers were not mutually exclusive.

Of 173 men who had ever performed ASE, 47% (n = 81) had ever felt something abnormal during ASE, with more than half of these men (63%, n = 50) reported the findings as a “lump”, while 38% (n = 30) reported bleeding and 28% (n = 22) reported ulcers and/or sores (Table 2). Most (66%, n = 115) found it easy or very easy to perform ASE. Only 8% (n = 13) found it difficult or very difficult to perform ASEs (Table 2).

Men were presented with potential resources and information that might be useful for MSM who had never performed ASEs (e.g. graphics on poster or website showing how to do an examination, having a doctor or nurse speak in person and explain how to do ASE, or online videos from a trusted source, see Supplementary Material). The top resource that men indicated would likely be helpful was an instructional graphics on posters or websites showing how to do ASE (66%, n = 113) (Table 3). Men were asked about information that might be useful to learn about anal ASE and syphilis such as how to do the examination, what a healthy anus should look, or what a syphilis lesion looks like (see Supplementary Material). Most men (63%, n = 106) reported all the information related to ASE (e.g. how to perform, a healthy look of anus, and syphilis), anorectal syphilis (e.g. what a syphilis lesion looks like, what a syphilis lesion feels like), other non-STI symptoms of the anus and symptoms of anal cancer would be helpful (Table 3).

**Table 3:** ASE positions, locations, resources, information among men, stratified by ever and never performing ASE.

	Ever performed* ASE (Number,Percentage)		Never performed ASE* (Number,Percentage)	
	N	%	N	%
**ASE positions**	**N = 176**		**N = 227**	
Standing	88	50.0	88	39.0
Squatting	80	45.0	61	27.0
Lying on the side	35	20.0	47	21.0
Lying on the back	36	20.0	31	14.0
Others	6	3.0	0	0.0
**Locations for performing ASE**	**N = 173**		**N = 232**	
Shower	106	61.0	151	65.0
Bed	40	23.0	66	28.0
Bathroom/toilet	85	49.0	105	45.0
Others	4	2.0	3	1.0
**Desired resources relating to ASE technique**^^^** (i.e., where to learn ASE, how to learn, from who to learn ASE)**	**N = 171**		**N = 328**	
Instructional graphics on poster or website	113	66.0	222	68.0
In-person explanation from doctor/nurse	82	48.0	172	52.0
In-person demonstration by a doctor/nurse using a training model/manikin	31	18.0	85	26.0
Videos of a doctor/nurse using a training model or a live person	64	37.0	162	49.0
Online videos from a trusted source (i.e. existing and available to general public)	81	47.0	182	55.0
Explanation from partners or friends	23	13.0	17	5.0
Do not need any resources	4	2.0	13	4.0
**Content of resources and information relating to ASE and anal syphilis**^^^** (i.e., what to look for in ASE, syphilis signs and symptoms, etc.)**	**N = 168**		**N = 325**	
How to do the examination	44	26.0	105	32.0
Where to do the examination	11	7.0	21	6.0
What a healthy anus should look like	43	26.0	106	33.0
What a syphilis lesion looks like	42	25.0	101	31.0
What a syphilis lesion feels like	42	25.0	105	32.0
What other sexually transmitted infections (STI) symptoms would look or feel like on the anus, including warts and herpes	0	0.0	80	25.0
Other non-STI symptoms of the anus (e.g., haemorrhoids)	31	18.0	75	23.0
Symptoms of anal cancer	32	19.0	80	25.0
All of the above	106	63.0	193	59.0

% Percentage.

*The two groups were not comparable as they were presented with different scenarios such as positions already used by men who had performed ASE vs positions likely to be used by men who had never performed ASE.

^^^Allowed multiple options and the answers were not mutually exclusive.

Among men who had ever performed ASE, 167 men answered the question about recommending ASE to other MSM, and 80% (n = 134) indicated that they were likely or very likely to recommend other MSM to perform ASE.

### Preferences that men who had never performed ASE likely to have if they were to perform ASE

Men who had never performed ASE were asked if they would consider ASEs regularly at the recommendation of a doctor to detect syphilis sores or ulcers. Among the 374 men who never performed anal self-examinations, 68% (250) would consider anal self-examinations in the future for anal syphilis detection, while 18% (65) would like more information about the examination. Nine percent (35) were unable to decide if they would consider anal self-examination and 5% (20) did not want to perform anal self-examinations at all.

Of the 374 men who had never performed ASE, 28% (n = 105) men engaged in receptive anal sex; 52% (n = 193) men engaged in versatile anal sex. Among 298 men engaging in receptive or versatile anal sex and never performed ASE, 69% (n = 205) would consider performing ASE to detect early syphilis in the future, while 18% (n = 54) would like to receive more information about ASE before making the decision. Eight percent (n = 24) were unable to decide if they would consider performing ASE, and 4% (n = 12) did not want to perform ASE at all.

Among the men recruited from the community and never performed ASE (n = 36), 44% (n = 16) indicated they would be willing to perform ASE, while 52% (n = 19) wanted more information or unsure about performing ASE in future (data not shown in “Results”) and 3% (n = 1) did not want to perform ASE in future.

Of the 250 men who were willing to consider performing ASE in the future regardless of their sex position, the median of the preferred frequency for ASE was two times per four weeks (IQR 1–4). Among these men, 205 men engaged in either receptive or versatile anal sex, and the median of the preferred frequency for ASE was four times per four weeks (IQR 1–4).

There were 227 men who had never performed ASE indicated they would most likely prefer standing (39%, n = 88) if they were to perform ASE in the future, followed by squatting (27%, n = 61) (Fig. 1).

Of the 232 men who answered the questions on locations they most likely to perform ASE, 65% (n = 151) reported the shower as the preferred location where they were likely to perform ASE, followed by bathroom or toilet (45%, n = 105) (Table 3). Almost half (43%, n = 98) would consider having their partner (regular romantic or regular sex partners) examine their anus when men were asked if they would let their partner examine their anus. Only a few (8%, n = 17) would consider a casual partner performing the examination.

When presented with choices of who they prefer to be performing an anal examination as a screening method, 45% (n = 53) of the 117 men who answered the question preferred to have a doctor performed an anal examination to check for syphilis lesions in their anus, although some men (28%, n = 33) preferred performing anal examination themselves and 27% (n = 31) did not have a preference.

Men were asked what type of information they would like about ASE in relation to technique of ASE, recognising abnormalities, and about anorectal syphilis. Of the 328 men answering questions, all of them would like at least some information about ASE and anorectal syphilis (e.g., how to perform ASE, how to distinguish between normal and abnormal findings, what a syphilis lesion looks like) with 59% (n = 193) wanting all the information about ASE, syphilis lesions, other non-STI symptoms of anus and symptoms of anal cancer (Table 3).

Men were also asked how they would like to access learning resources for ASE such as learning from online platform, a video, or a doctor. The majority (96%, n = 315) of men who had never performed ASE would like to learn from various resources such as through a website with instructional graphics, online videos, through doctors/nurses, and a small number reported willing to learn from friends or partners, whilst only 4% (n = 4) reported not needing any resources (Table 3).

Of the 324 men who answered questions on future ASE considerations and had never performed ASE, the majority were very likely or likely to consider performing ASE in the future (89%, n = 288), whilst 2% (n = 6) chose not to perform ASE in the future, and 9% (n = 20) were unable to decide.

## Discussion

Our study shows that about two-thirds of MSM who participated in the survey had never performed ASE, although the majority (88%) of these men were interested in performing ASE in the future for detecting anal syphilis lesions and most preferred to do it once per month. Men who had ever performed ASE largely found it easy to perform ASE, and most would recommend performing ASE to other MSM. Although various ASE positions were reported, the two groups of men who had ever performed and who had never performed ASE uniformly preferred the standing position, followed by the squatting position. Men in both groups preferred performing ASE in the shower compared to other locations. Most men would like to learn or gather more information about how to perform ASE on more accessible forms of learning such as videos, graphics on a poster, and a website instead of in-person training such as a doctor or nurse demonstrating face-to-face training. Men also expressed wanting to get more information about syphilis, especially anal syphilis and the abnormalities that likely to encounter during ASE. There was no difference between the two groups in terms of age, HIV status and PrEP use, implying that future public health campaigns should target everyone rather than a specific population.

The positive attitudes towards ASE for syphilis detection reported here are consistent with our previous qualitative study of MSM on ASE^21^. Similarly, the study by Ong et al*.* showed a high level of acceptance towards performing ASE for anal cancer screening^20^. The ongoing syphilis epidemic among MSM, highlight the need for novel strategies for the early detection of syphilis that will reduce the duration of infectiousness^5^.

Before proceeding to implement ASE, it would be first important to establish that men could detect primary syphilis lesions in the anal canal. If shown to be effective, ASE for syphilis detection would be a new concept and intervention for MSM; educating MSM on performing ASE correctly would be required. Most men preferred this information to be publicly available on a poster or website rather than gathering this information from health professionals.

Before implementing such a program, it would also be important to determine how commonly men presenting with findings that were not primary syphilis or other conditions of concern. This would be an important consideration because it would influence the cost and cost-effectiveness of any such program. If this was very common, then it may limit the usefulness of such a program.

There are several limitations to the study. First, most of the participants were recruited from a sexual health clinic, and only a small proportion was recruited from the community. Furthermore, only 20% of the clinic attendees who were sent the survey completed the survey. Therefore, we might not be able to generalise the finding to the whole MSM community, as we did not compare the demographics of participants who completed the survey to the overall clinic attendees who were invited but did not take part in the survey. Moreover, men attending a sexual health clinic may be more health-conscious and perhaps more willing to perform ASE compared to men who did not attend a sexual health clinic. Among the men recruited from the community and never performed ASE, almost half (44%) indicated willingness to perform ASE compared to 68% of sexual health centre attendees. Over half (52%) of the men recruited from the community wanted more information or were unsure about ASE implying that more health promotion and education about ASE might be required if ASE were to be recommended in the future. Second, the survey was limited to participants from Australia to minimise spam responses from outside of Australia, and therefore the findings can only apply to MSM residing in Australia. Third, we did not know why 4% of men reported that they would not consider performing ASE in the future, as this information was not collected. Finally, the survey had a low participation rate which may have biased the responses, particularly for example towards the percentage of men who had previously performed ASE.

## Conclusion

This study found that most men who had never performed ASE are willing to perform ASE to detect any abnormalities in the future. If ASE is shown to be effective and cost-effective in detecting early anal syphilis lesions, this could be translated into a public health intervention entailing ASE combined with regular STI screening.

## Supplementary Information

Supplementary Information.

## Data Availability

The datasets used and/or analysed during the current study are available from the corresponding author, on reasonable request, with the permission of the Alfred Hospital Ethics Committee. The datasets can be requested by a researcher who provide a methodologically proposal.
