# Supplementary material for: A cross-sectional survey on attitudes of men who have sex with men towards anal self-examination for detection of anal syphilis
Source: Sci Rep. 2022 May 27;12:8962. doi: 10.1038/s41598-022-12881-3 (PMC9142515; doi:10.1038/s41598-022-12881-3)
Supplement: Supplementary file 1 — Supplementary Information. [file 41598_2022_12881_MOESM1_ESM.pdf]

SELF-EXAMINATION FOR ANAL SYPHILIS SURVEY (SEAS-S)

First page

Consent Page

We would like to invite you to participate in an online survey about anal self-examination, it will take about 10 minutes to complete.

Participation in this survey is voluntary and no personally identifiable information will be collected in this survey. Your responses to this survey will remain confidential to the MSHC research team.

This study is approved by the Alfred Hospital Ethics Committee (Project xx/20). A detailed Participant Information Sheet can be downloaded here -> [Link]

If you agree to participate in this survey, please click on the 'Agree' button below.

If you do not want to participate in this survey, please click on the 'Disagree' button below. (Forced response)

- Agree
- Disagree

Second page – a brief description of anal examination

1. **Introduction**

You are invited to take part in this survey on self-examination for anal syphilis detection.

What is anal self-examination (ASE)?

Anal self-examination means inserting your finger (usually index or pointing finger) into your anus and feeling around the anal canal as far as you can while twisting your finger all the way around to feel all sides (360°). Your anal canal (2-5 cm in length) should normally feel smooth. You can also use a mirror to check the anus and surrounding area for any abnormalities.

In this survey, we would like to determine how men feel about performing anal self-examination.

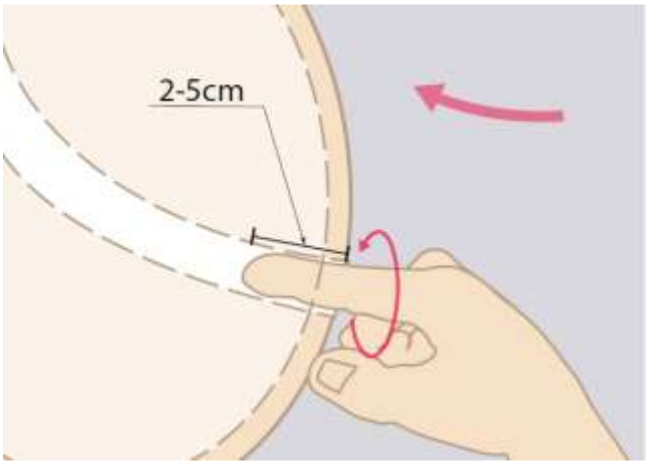

Diagram showing how to perform anal self-examination

What is syphilis?

Syphilis is a sexually transmitted infection (STI) and can be treated effectively with antibiotics. If you want to know more about syphilis, read here: syphilis

Demographics

1. What is your age?
2. Are you currently living in Australia?
   - Yes
   - No (end of survey)
3. What sex were you assigned at birth (i.e. what was specified on your original birth certificate?)
   - Male
   - Female (end of survey)
4. Which of the following best describes your current gender identity?
   - Male
   - Female (end of survey)
   - Non-binary/gender fluid
   - Different identity, please specify_____
5. Which of the following best describes your sexual practice?
   - Sex with women only (end of survey)
   - Sex with men only in the last 12 months
   - Sex with both men and women in the last 12 months
   - Prefer not to say
6. What is the highest degree or level of education you have completed? If currently enrolled, highest degree received.
   - No schooling completed

- Some secondary education - Years 9 and below
- Secondary education completed
- Certificate I,II,III,IV level
- Advanced diploma and diploma level
- Bachelor’s degree
- Master’s degree/graduate diploma/graduate certificate
- Postgraduate degree

7. What is your usual sexual position during anal sex with men?
    - Top (you insert your penis into your sexual partner’s anus)
    - Bottom (your sexual partner inserts his penis into your anus)
    - Versatile (both top and bottom)
    - Only oral sex

8. What is your HIV status?
    - Positive (jump to Q10)
    - Negative
    - Prefer not to say
    - I do not know

9. Are you currently taking pre-exposure prophylaxis (PrEP) to prevent HIV?
    - Yes
    - No

10. Have you ever been diagnosed with syphilis?
    - Yes
    - No
    - I don’t know / I don’t remember

Anal self-examination

11. Have you ever self-examined your anus (i.e. insert your finger into your anus in order to check for abnormalities, as described in the introduction)?
    - Yes (carry forward choices, Q 12)
    - No (carry forward choices, Q 22)

Currently practising ASE

12. How frequently do you do anal self-examination?
    - (xxx times per week/month/year)

13. Which position/s do you usually use for anal self-examination? Please select all apply
    - Pictures below – label – lying on the side, squatting and standing on a stool or toilet
    - Other positions (free text)
    -

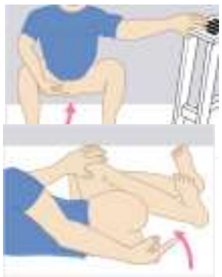

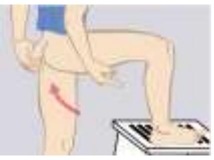

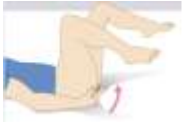

14. Do you usually use the following items to perform anal self-examination? Please select all apply
    - Gloves
    - Lubricant
    - Soap
    - Mirror
    - Water
    - Others: free text
    - None of the above (exclusive choice)

15. Where do you usually perform anal self-examination?
    - In shower
    - In bed
    - In bathroom/Toilet
    - Others: free text

16. Have you ever felt something in your anus during a self-exam that made you worry about your health?
    - Yes
    - No (carry forward choices, Q 18)

17. Please describe what you felt in your anus that made you worried for your health. If this has happened more than once please describe what you felt the last time this happened. (multiple options)
    - lumps
    - Ulcers or sores
    - Bleeding
    - Others: Free text

18. How do you find performing anal self-examination?

(Scale 1 to 5, horizontal)

- Very easy
- Easy
- Neutral
- Difficult
- Very difficult

19. What kind of resources do you think are helpful for other MSM to learn how to do an anal self-examination? (multiple options)
    - Graphics on poster or website showing how to do an examination
    - Having a doctor or nurse speak to me in person and explain how to do it
    - Having a doctor or nurse using a training model/manikin in person to explain how to do it
    - Videos of a doctor or a nurse using a training model or a live person
    - Online videos from a trusted source (e.g. www.anal.org.au)
    - From partners or friends
    - I don't need any resources

20. What information do you think will be helpful for other MSM to learn about anal self-examination? (multiple options)
    - How to do the examination
    - Where to do the examination
    - What a healthy anus should look
    - What a syphilis lesion looks like
    - What a syphilis lesion feels like
    - What other sexually transmitted infections (STI) symptoms would look or feel like on the anus, including warts and herpes
    - Other non-STI symptoms of the anus (e.g. haemorrhoids)
    - Symptoms of anal cancer
    - All of the above (exclusive response)

21. If a doctor or a nurse recommended anal self-examination for detecting syphilis chancre or ulcers or sores in the anus, how likely will you recommend other MSM to practise anal self-examination? (Carried forward choice, Q 31) (Scale 1-5)

    1: Very likely
    2: Likely
    3: Maybe
    4: Unlikely
    5: Very unlikely

Never done ASE

22. Will you consider performing regular anal self-examinations in the future if a doctor advised you to start doing anal self-exam regularly so that you could detect syphilis ulcers or sores? (forced response)
    - Yes (carry forward choices, Q 23)
    - No (Skipped logic, end of survey)
    - Undecided or I cannot make decisions now (carry forward choices, Q 28)
    - I want more information on how to do anal self-examination first (carry forward choices, Q 28)

23. How often will you consider doing an anal self-examination?
    - (xxx times per week/month/year)
    - As much as was recommended by the health professionals

24. Please rank the positions below from 1 to 4 (1 being the position you would be most comfortable or convenient to perform the exam? (multiple options/rank options)
    - Pictures below – label – lying on the side, squatting and standing on a stool or toilet

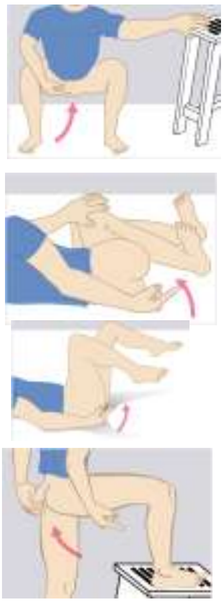

25. Where would you prefer to do anal self –examination?
    - In shower
    - In bed
    - In bathroom/Toilet
    - Others: free text

26. Would you ever consider having your partner perform an anal exam on you?

- Yes, casual partner (e.g. anonymous partners, one-night-stand partners) (carried forward choice 28)
- Yes, regular romantic partner (e.g. husband, boyfriend) (carried forward choice 28)
- Yes, regular sex partners (e.g. friends with benefits, fuckbuddies)
- No (carried forward choice 27)

27. Who would you prefer performing anal examination for detecting syphilis ulcers/sores?
    - myself
    - a doctor
    - I don't have any preference

28. What kind of resources would you want to use to learn how to do anal self-examination? (multiple options)
    - Graphics on poster or website showing how to do an examination
    - Having a doctor or nurse speak to me in person and explain how to do it
    - Having a doctor or nurse using a training model/manikin in person to explain how to do it
    - Videos of a doctor or a nurse using a training model or a live person
    - Online videos from a trusted source (e.g. www.anal.org.au)
    - From partners or friends
    - I don't need any resources

29. What information would you like in learning about anal self-examination? (multiple options)
    - How to do the examination
    - Where to do the examination
    - What a healthy anus should look
    - What a syphilis lesion looks like
    - What a syphilis lesion feels like
    - Sexually transmitted infections (STI) symptoms on anus including other STI such as warts and herpes
    - Other non-STI symptoms of the anus (e.g. haemorrhoids)
    - Symptoms of anal cancer
    - All of the above (exclusive choice)

30. If a doctor or a nurse recommended anal self-examination for detecting syphilis chancre or ulcers or sores in the anus, how likely will you start doing it?

    (Scale 1-5)
    1: Very likely
    2: Likely
    3: Maybe
    4: Unlikely

5: Very unlikely

31. Which of the following scenario would you choose for regular anal self-examination for syphilis? (Discrete choice experiment) (end of survey)

Table 1: Example of choice set

	A	B	C
Frequency	Once a week	Once a year	I do not prefer options A or B
STI screening frequency	Once every 6 months	Once every 3 months	
Support if abnormality found	See nurse	See sexual health doctor/nurse	
Speed of getting support	3 days	Same day	
Instructions	Online video	Demonstration by doctor on yourself during a consultation	
Cost	No gloves, soap as lubricant	Free gloves, lubricant	
Reminder	Email every 3 months	SMS once a year	
Accuracy	100% early syphilis detected	25% early syphilis detected	

Table 2: The above choice set may alter using the different levels here

Attributes	Levels
Recommended frequency of examination	Once a week<br>Once a month<br>Once every 3 months<br>Once a year
STI screening frequency	Once every 6 months<br>Once every 3 months<br>When symptoms of STI develop
Support if abnormality found	See sexual health doctor<br>See GP<br>See nurse<br>Online chat
Speed of getting support	Same day<br>3 days<br>7 days
Instructions for performing self-examination	Online video<br>Demonstration by doctor on yourself during a consultation<br>Verbal explanation by doctor<br>Written instructions
Reminder system to do examination	SMS<br>Email<br>Phone call<br>Reminder message from phone app

Accuracy of picking up abnormality using self-examination	100% early syphilis detected<br>75% early syphilis detected<br>50% early syphilis detected<br>25% early syphilis detected
